# New Appliances and Things Medical

**Published:** 1909-08-07

**Authors:** 


### NEW APPLIANCES AND THINGS MEDICAL.

**[We shall be glad to receive at our Office, 28 & 29 Southampton Street, Strand, London, W.C., from the manufacturers, specimens of all new preparations and appliances.]**

### "VINDEVIE."

Juice of Grapes.

Sole Importers, Messrs. Schweppes, Ltd.

The product before us—"Vindevie"—is an unfermented wine and consists of grape juice treated by a special process. This process consists essentially in a combination of pressure and sterilisation. The product contains none of the chemical preservatives which are used so freely in the preparation of the various kinds of fruit-juice cordials; it further contains no alcohol and no excess of sugar, as in the many fruit-syrups on the market. "Vindevie," either diluted with twice its volume of water or soda water or undiluted, has an exceedingly clean, refreshing, and pleasant taste. It contains all the constituents of good grape-juice, and hence is available for all the therapeutic uses of grape-juice, such, for instance, as constipation, anæmia, and those blood conditions in which the exhibition of the organic and inorganic salts of grape-juice is indicated. We are pleased to have received a sample of this beverage, and we think it can well be recommended by the medical profession, and has a very extensive sphere of usefulness before it.

### "CHELTINE" FOODS.

(Cheltine Foods and Chocolate Co., Cheltenham.)

We have received several samples of these foods—*e.g.* brown-meal biscuits, almond biscuits, cocoanut biscuits, meat biscuits, and white-meal biscuits, also "Cheltine" diabetic food, consisting of a powder with a biscuit-like taste. Various domestic receipts are given for the utilisation of "Cheltine" food in ordinary cookery. We have also received a notice of a new artificial sweetener in liquid form made by the same manufacturers, to which the name "Porcherine" is given. This latter substance is capable of entirely replacing sugar in the dietary. The London agent for all these preparations is Harrods, Ltd. The "Cheltine" foods and preparations are intended for patients suffering from diabetes or glycosuria. They, however, contain starch in such a form as to give the usual iodine reaction, but it is claimed for them that they can be taken by diabetics without increasing the sugar of the urine. The samples are accompanied by clinical evidence confirming the value of these preparations in cases of diabetes. The taste of the biscuits and the powder is quite pleasant, and we think the products are to be recommended, but in cases of severe glycosuria or true diabetes it will be necessary for the medical man to watch carefully their effect upon the excretion of glucose.

